# Adaptation of a Live Video Mind–Body Program to a Web-Based Platform for English-Speaking Adults With Neurofibromatosis: Protocol for the NF-Web Study

**DOI:** 10.2196/27526

**Published:** 2021-06-10

**Authors:** Ethan Gabriel Lester, Sarah Whitall Hopkins, Paula Jean Popok, Ana-Maria Vranceanu

**Affiliations:** 1 Integrated Brain Health Clinical and Research Program Department of Psychiatry Massachusetts General Hospital Boston, MA United States; 2 Harvard Medical School Boston, MA United States; 3 Suffolk University Boston, MA United States

**Keywords:** neurofibromatosis, quality of life, stress management, mind–body, asynchronous delivery, resiliency, mobile phone

## Abstract

**Background:**

Neurofibromatosis (NF) is a rare genetic condition associated with lower but modifiable quality of life (QoL). Although a virtual live video program (Relaxation Response Resiliency Program for Neurofibromatosis [3RP-NF]; efficacy randomized controlled trial underway) that we created has been made available, ongoing barriers impede some patients from engaging in this intervention. A necessary next step is to develop a stand-alone web-based intervention that reduces barriers to accessing NF-specific psychosocial care.

**Objective:**

First, we aim to develop a web-based platform (Neurofibromatosis-Web [NF-Web]) of our mind–body resiliency program (3RP-NF) through qualitative interviews with participants from an adult efficacy randomized controlled trial. Second, we aim to iteratively optimize the feasibility, acceptability, credibility, and satisfaction of the NF-Web platform through open pilot trials with participant exit interviews and explore quantitative outcomes within this sample. Here, we describe the protocol and study design, intervention, and analysis plan.

**Methods:**

For aim 1, we will invite completers from our efficacy trial to participate in qualitative interviews. We will use data from these interviews to adapt the content of the live video program for asynchronous delivery and understand how to create a user-friendly format for an engaging web platform. For aim 2, we will enroll eligible participants recruited for the efficacy trial who could not enroll because of treatment barriers. Eligible participants will complete QoL, depression, anxiety, pain, treatment satisfaction, and program credibility measures at baseline and posttest. Inclusion criteria are identical to those for the efficacy trial, including stress and coping difficulties (self-report), no change in antidepressant medication in the past 3 months, no psychotherapy in the past 3 months, no major upcoming surgeries in the next 12 months, English speaking, ability to complete questionnaires on the web and participate in live video interventions, and consent before participation. The primary outcomes are feasibility, treatment satisfaction, and credibility. The secondary outcomes include physical, psychological, social, and environmental QoL; depression; anxiety; pain intensity; and pain interference. We will enroll at least two group cohorts and iteratively refine the program based on participant feedback after each cohort completes the open pilot trial.

**Results:**

This trial is ongoing. We have completed the interviews (n=23) and analyzed the data to construct the website. Afterward, we will recruit our cohorts for the trial (approximately n=15/cohort; total=30). Recruitment will end by May 2021, with plans to analyze the data by October 2021.

**Conclusions:**

We will develop the first web platform for people with NF with difficulties managing stress and NF symptoms and report on feasibility and preliminary effects in improving QoL and psychosocial functioning. NF-Web has potential to extend the reach of our 3RP-NF intervention by removing barriers to care, including lack of trained providers, scheduling difficulties, and appearance concerns.

**International Registered Report Identifier (IRRID):**

DERR1-10.2196/27526

## Introduction

### Background

Patients with neurofibromatosis (NF; NF1, NF2, or schwannomatosis) have lower but modifiable quality of life (QoL) [1], higher depression [2], anxiety [2], pain [1], stress, and social isolation [3] compared with the general population [4]. Given that NF is incurable, and current medical treatments have limited ability to eliminate symptoms, addressing modifiable psychosocial factors through tailored psychosocial interventions is essential to improve QoL in this population. Despite this need, patients with NF do not have access to tailored, in-person evidence-based psychological care because of barriers such as distance, cost, time or scheduling, and lack of trained providers. Our team at Massachusetts General Hospital used mixed methods approaches to adapt an evidence-based mind–body program for the specific needs of adults with NF (Relaxation Response Resiliency Program for Neurofibromatosis [3RP-NF]; NF1, NF2, or schwannomatosis) [3], adolescents with NF (Resilient Youth with NF) [5], and adults with NF2 who are deaf (Relaxation Response Resiliency Program for NF2) [6] for virtual delivery to bypass these barriers to care. In three single-blinded pilot randomized controlled trials (RCTs) [3,5,6], we have shown that these programs are feasible, acceptable, and superior to an attention placebo health education counterpart in improving QoL, pain, and psychosocial functioning. With funding from the United States Department of Defense (DoD), we are currently conducting fully powered efficacy trials of the 3RP-NF intervention in adults (aged 18 years or older) and adolescents (aged 12-17 years).

Although virtual delivery increased the reach of our intervention and allowed the recruitment of geographically diverse individuals, ongoing barriers impede all people with NF from enrolling. Our ongoing DoD-funded RCT receives interest from patients across the globe; yet, time-zone differences (eg, a difference of 10.5 hours between EST and Indian Standard Time); occupational or family obligations; limited and convenient scheduling (eg, 10 PM local time, weekend scheduling); 8-week, 90-minute weekly scheduling; and severe actual or perceived appearance concerns (eg, cutaneous and plexiform neurofibromas with facial tumors, palsy, café au lait spots, freckling) cause people to decline participation (approximately 30% who are contacted) [7]. As there is a lack of trained providers to administer evidence-based psychosocial care, a web-based platform (ie, a program hosted on a website server and created using HTML) in addition to the live videoconferencing program allows for asynchronous delivery of the intervention and could bypass these barriers to participation.

### Objective

The purpose of this study is to develop the first web-based platform of a mind–body program for adults with NF with stress and difficulties in managing NF symptoms. Our first aim (aim 1) is to adapt the adult mind–body program, 3RP-NF, for web-based delivery using qualitative data from semistructured interviews with participants who completed the efficacy trial. To achieve this aim, we will collect information about the participants’ experiences, perceptions of a web-based platform, and willingness to participate. We will use this information to develop a web-based platform—Neurofibromatosis Web (NF-Web)—that will be designed to be compatible with desktops, laptops, and mobile devices (eg, smartphones and tablets). We expect that participants from the efficacy trial will be willing to participate in semistructured interviews and provide meaningful feedback to inform the adaptation of the 3RP-NF intervention for web-based delivery. Our second aim (aim 2) is to optimize NF-Web through at least two subsequent open pilot studies with exit interviews. For each open pilot, we aim to optimize the feasibility and acceptability of NF-Web and explore the quantitative outcomes. We hypothesize that the final iteration of NF-Web will meet the a priori set feasibility and acceptability benchmarks [8-10]. We also hypothesize that participation in NF-Web will be associated with improvement in quantitative outcomes from baseline to posttest.

Related to the aims and scope of this pilot study, we will not be testing the use of different technologies for NF-Web delivery (eg, mobile and tablet vs desktop or laptop modalities); nor are we directly interested in the acceptability of such technologies (eg, the advantages of the website over the live video program). Rather, we are interested in studying the psychosocial program itself as a web-based tool to deliver the mind–body program to participants who typically have a higher burden when accessing psychosocial care. The creation of NF-Web essentially involves taking an efficacious program, which is delivered either in person or through live video by a trained clinical psychologist, and making it more accessible by delivering it through a web-based platform. Here, we describe our methodology for both the study aims.

## Methods

### Study Design

**Figure 1:**
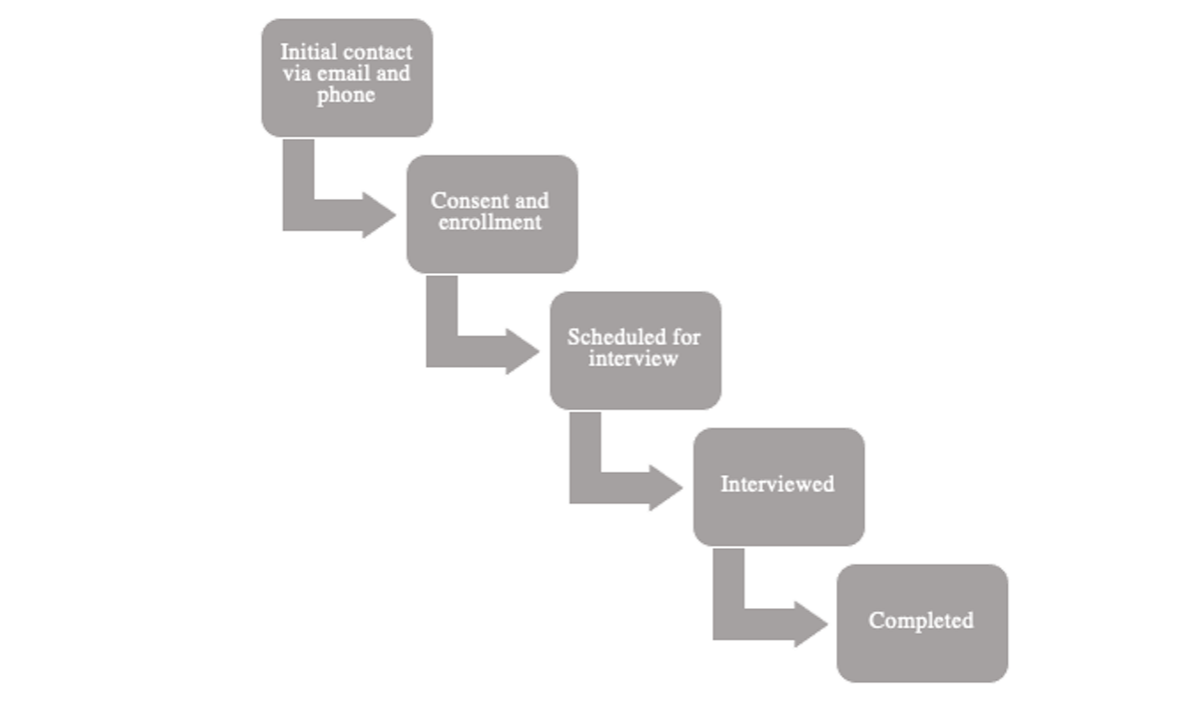
Qualitative study design.

**Figure 2:**
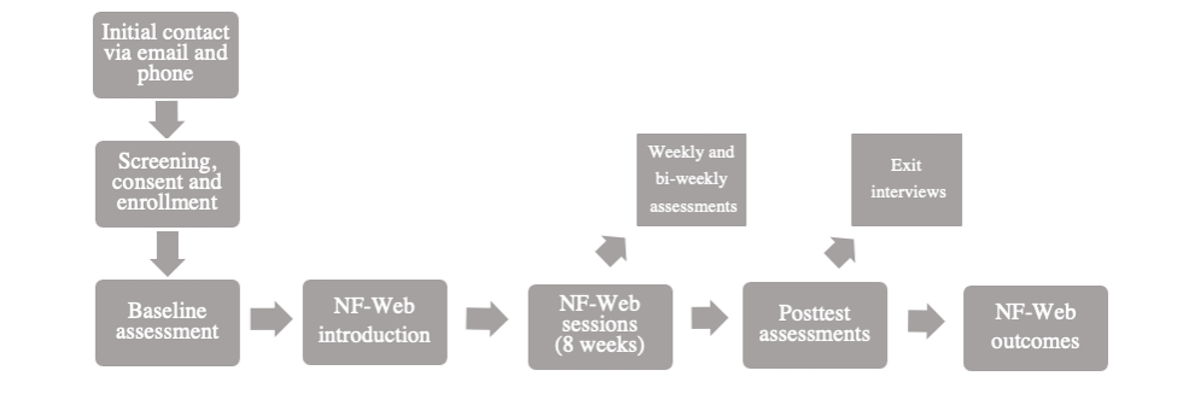
Open pilot study design. NF-Web: Neurofibromatosis-Web.

For aim 1, we will conduct individual qualitative semistructured interviews to adapt the 3RP-NF intervention for web-based delivery. For aim 2, we will conduct at least two subsequent open pilot studies of NF-Web to optimize feasibility and acceptability and explore changes in the quantitative outcomes (Figures 1 and 2). We will modify NF-Web after each open pilot to maximize feasibility and acceptability, as needed.

### Setting

Studies related to both aims 1 and 2 will be conducted at a large northeastern academic medical center in the United States. Our use of virtual recruitment and web-based intervention delivery allows participants from the United States as well as the rest of the world to engage in the study at their convenience (eg, location, time of day, pace). We will recruit participants through our research tracking log that contains both efficacy trial completers (assessments complete at 1 year time point; aim 1) and participants who expressed interest in the 3RP-NF live video program but could not participate because of treatment barriers (aim 2).

### Inclusion and Exclusion Criteria

The inclusion and exclusion criteria for this study are identical to those listed in the 3RP-NF trial [11] and are outlined in Textbox 1. The participants completed the study screening over live videoconferencing with the study staff. We assessed stress using the 4-item Perceived Stress Scale [12] (score≥6) [13].

**Study inclusion and exclusion criteria.**

**Inclusion Criteria**

- Diagnosis of neurofibromatosis (NF; NF1, NF2, or schwannomatosis)
- Adults aged 18 years or older
- Full understanding of the informed consent process, study procedures, and assessments in English
- ≥Sixth grade reading level (self-report)
- Difficulties coping with NF symptoms (self-report)
- Score≥6 on the 4-item Perceived Stress Scale

**Exclusion Criteria**

- Major medical comorbidity, not NF-related, expected to worsen in the next 12 months
- Change in antidepressant medication (within past 3 months)
- Recent participation in cognitive behavioral therapy or relaxation therapy (within past 3 months)
- Has diagnosis of significant mental health conditions requiring immediate treatment (eg, untreated bipolar disorder, psychotic disorder, active substance dependence) by self-report and observation during prescreening
- Unable or unwilling to complete assessments electronically through REDCap (Research Electronic Data Capture; Vanderbilt University)
- Unable or unwilling to participate in web-based, self-guided sessions

### Recruitment

For aim 1, we will first email participants who were randomized to the 3RP-NF intervention and completed the last assessment point of our efficacy trial and invite them to participate in a live video qualitative interview with a trained clinical psychologist with expertise in NF (50 min). We will use purposive sampling to achieve population-consistent representation of the three NF types (NF1, n=10; NF2, n=6; and schwannomatosis, n=4). We will recruit more participants if we do not achieve theme saturation. If participants meet enrollment criteria and are interested in participating, they will receive the consent form to review and return through secure email. After obtaining consent, we will schedule the participants for an interview.

For aim 2, we will recruit participants (n=30; approximately 15 per cohort) for the open pilot phase of NF-Web. These will be participants who previously expressed interest in participating in the live video program but ended up not participating because of treatment barriers. The study staff will describe the trial, and all participants will be screened by the study staff over the phone. The screening procedures are the same as those outlined in the 3RP-NF efficacy trial protocol [11].

### Vidyo Software for Interviews

We will conduct the semistructured interviews using the secure, Health Insurance Portability and Accountability Act–approved, live videoconferencing software, Vidyo (Vidyo, Inc). After obtaining consent, we will email the participants the instructions to download, install, and access Vidyo on their personal webcam-equipped, internet-connected devices (eg, laptop and desktop computers, tablets). We expect many participants to have had the software installed for their previous efficacy trial participation. We will make telephone appointments available for those needing assistance with installing the software.

### Screening and Enrollment

The clinical research coordinator (CRC) will obtain informed consent and ask the participants to return the signed consent form electronically through secure email. Participants are considered enrolled in the study when signed consent is received. The CRC will schedule a time with each enrolled participant for their individual semistructured video interview. We will provide an additional overview of the study procedures at the beginning of each interview. The study interviewer will ensure that the participants understand the study procedures and that we will audio record the interview. The participants will be interviewed on (1) experiences of living with NF; (2) experience during the live video program (ie, efficacy trial); (3) attitudes and perceptions of NF-Web, including participation barriers and facilitators; (4) perceptions about modifying session content and skills; and (5) considerations for decreasing barriers to participation. We will save audio on a secure and password-protected computer.

For aim 2, we will recruit participants who could not participate in the efficacy trial using the tracking log for our efficacy RCT. We will email or call the potential participants. We will screen participants over the phone to ensure that the eligibility criteria are met. The research staff will cover important components of the study protocol, and we will ask eligible participants to return the informed consent document to the CRC electronically through secure email.

### Treatment Conditions

#### 3RP-NF Program

We anticipate that the core content of the 3RP-NF intervention [11] will remain the same, with modifications focused primarily on content delivery and participant engagement. Briefly, the 3RP-NF intervention combines mind–body skills, including relaxation response elicitation with cognitive behavioral approaches and perspectives from positive psychology, into a virtual, multimodal intervention to increase coping strategies for those dealing with NF symptoms and associated stress. Core elements of the original 3RP-NF intervention include (1) relaxation response practice (eg, breath awareness, body scan, meditation, and mindfulness), (2) cognitive behavioral coping skills (eg, enhancing awareness of the connection among emotions, thoughts, and behavior), (3) positive psychology skills (eg, appreciation, empathy, and humor), and (4) healthy behaviors (eg, diet, exercise, and sleep). The session titles from the adult efficacy trial are presented in Textbox 2 [11].

**Session outline of Relaxation Resiliency Response Program for Neurofibromatosis and the web-based platform: session week and respective session title.**

**Week 1**

- Symptom management, stress management, and resiliency training

**Week 2**

- The relaxation response

**Week 3**

- Stress and symptom awareness for patients with neurofibromatosis

**Week 4**

- Mending mind and body of patients with neurofibromatosis

**Week 5**

- Creating an adaptive perspective

**Week 6**

- Promoting positivity

**Week 7**

- Healing states of mind

**Week 8**

- Humor, empathy, and staying resilient

#### NF-Web Platform and Procedures

NF-Web is a website version of the 3RP-NF program [11]. We anticipate that NF-Web will have 8 sessions (Textbox 2) with content presented in multiple modalities (eg, written, audio, video, interactive components) and administered entirely on the web without a live clinician.

After participants provide consent and complete baseline assessments, we will send them an email with detailed information on how to begin the web-based intervention (ie, *NF-Web Account Setup* email). NF-Web will require a secure log-in to access content with a username assigned by the study staff (eg, MGHNFW01) and a unique password. The CRC will keep a log of all usernames and passwords on a password-protected computer. We will give participants website instructions through email and review the terms and conditions of use provided on the website, which will include limits to confidentiality (risks similar to usual internet use; disclosure at their discretion) and ways to protect their information. The study staff will be available to answer all participant questions and concerns during the initial account creation and setup for the program.

After enrollment, participants will log in to NF-Web and progress through the web-based program at their own pace for 8 weeks (streaming video content embedded from YouTube; no software download required). Each week, participants will view session content consisting of mind–body video modules, audio recordings, written text, quizzes, and homework assignments (submitted through email or the web platform) from the 3RP-NF program. The participants will have access to completed session content from previous weeks as they await the following week’s session after completing the weekly assignments (viewing videos, completing in-session exercises and quizzes). In addition, participants will be informed of the optional discussion board where they can directly post content (deidentified for the trial owing to institutional review board requirements; data used to potentially assess user experience and engagement ethnographically). The participants will also have the option (ie, opt in or out) at the beginning of the program to receive daily or weekly sessions and skill practice reminders through email and text messages. Sessions will be unlocked by the study staff and administered weekly to the participants to pace their learning and practice (ie, new content available each week). We will have 2 cohorts of participants for each round (2 rounds of approximately 15 each, n=30) to attempt simulation of the NF-Web platform components (eg, discussion board use and monitoring, administrative tasks). The treatment fidelity for NF-Web mirrors that of the efficacy trial [11], with the addition that all materials for NF-Web will be prerecorded. At any point during the study, the participants will be able to ask nonurgent questions by sending an email to the study staff through a secure website form on NF-Web. This approach to the website may be modified or adapted before being finalized based on participant feedback gathered in the aim 1 exercise.

### Considerations for Participant Safety During a Virtually Delivered Program

Participant safety is evaluated at multiple study points. Participants complete the Patient Health Questionnaire (9-item version) [14] to measure depressive symptomatology at baseline and posttest, which includes a question related to suicidality. If a participant reports thoughts of self-harm or suicidality, the CRC and principal investigator (PI) are notified, and the PI will contact the participant within 24 hours to conduct a risk assessment. Safety is always prioritized over study participation. If it is determined that participants need a higher level of care, they will be provided with information about resources for care, as appropriate. Participants who do not require higher levels of care may continue in the study and are monitored throughout the posttest. The same risk-related assessments are conducted once more at the posttest, and the same response procedure is followed.

### Assessments

For aim 1, we will conduct participant interviews through a secure video platform, Vidyo, and audio record and code qualitative content. Participant demographic data will be available from previous participation in the efficacy trial. The interview data will be used to develop and refine the NF-Web platform.

For aim 2, participants will complete web-based surveys through REDCap (Research Electronic Data Capture) [15], including reliable and valid measures. All measurements are presented in Table 1 and directly mirror the efficacy trial, with the exception of weekly or biweekly assessments throughout the NF-Web program.

**Table 1:** Neurofibromatosis-Web program measures and assessment time points.

Construct	Measure or description	Outcome	Time point
Demographic	Demographics include age, gender, race, education, yearly income, and marital status.	Descriptive	Baseline
Quality of life	The WHOQOL-BREF^a^ [16] assesses subjective quality of life in 4 domains: physical, psychological, social, and environmental.	Primary	Baseline, posttest
Depression	The Patient Health Questionnaire for Depression-9 [14] assesses depressive symptoms and severity consistent with *DSM-5*^b^ criteria of major depression.	Secondary	Baseline, posttest
Anxiety	The Generalized Anxiety Disorder Scale-7 [17] assesses anxiety symptoms and severity consistent with *DSM-5* criteria of generalized anxiety.	Secondary	Baseline, posttest
Pain intensity	The 11-point Numeric Rating Scale [18] assesses intensity of pain experience.	Secondary	Baseline, posttest
Pain interference	The Brief Pain Inventory [19] pain interference subscale assesses intrusion of pain experience on daily functioning.	Secondary	Baseline, posttest
Quality of life (abbreviated)	The WHOQOL-BREF [16] (abbreviated items) assesses subjective general quality of life and quality of life satisfaction with two items.	Exploratory	Weekly
Depression (brief)	The Patient Health Questionnaire-2 [20] assesses frequency of depressed mood and anhedonia.	Exploratory	Biweekly
Anxiety (brief)	The Generalized Anxiety Disorder-2 [21] assesses frequency and severity of generalized anxiety symptoms.	Exploratory	Weekly
State affect	The Positive and Negative Affect Scale–Short Form [22] assesses state positive and negative affect.	Exploratory	Weekly
Website user experience	User Experience Questionnaire [23] assesses patient overall experience using the website.	Exploratory	Weekly
Treatment satisfaction	The Client Satisfaction Questionnaire [24] assesses patient satisfaction with the web-based program.	Primary	Posttest
Treatment credibility	The Credibility Questionnaire [25] assesses how believable, convincing, and logical patients perceive the web-based program to be.	Primary	Posttest

^a^WHOQOL-BREF: World Health Organization Quality of Life Instrument, Short Form.

^b^*DSM-5*: *Diagnostic and Statistical Manual of Mental Disorders-5*.

We will send the participants a secure link through REDCap [15] to complete baseline assessments within a week of beginning the intervention. If a consented participant does not complete the questionnaires after a week of being sent the link, we will contact the participant and assist them in completing the questionnaires over the phone. The participants will also be sent weekly surveys throughout the program with less strict enforcement of assessment adherence owing to the aim of feasibility testing and because these time points do not affect primary or secondary outcomes. The participants will be emailed a reminder within 24 hours of the final session to complete the posttest assessments, and the CRC will call within three days if they have not completed the assessments. If a participant does not complete the posttest measures, the PI will continue contact attempts through phone and/or email as needed within two weeks of completing the last session. Any participants who are unreachable by the second full week (ie, 14 days) after posttest administration will be deemed lost to follow-up. We will conduct exit interviews lasting approximately 15 minutes at the participants’ convenience to gather feedback on their participation, including suggestions for program improvements, barriers and facilitators of program engagement, and the internet device they used to access the program (eg, mobile and tablet vs desktop).

### Data Analysis

For aim 1, we will transcribe and analyze qualitative data from participants using NVivo 10 qualitative data analysis software (QSR International) [26]. Each interview will be transcribed and coded after the collection. We will use a thematic content analysis informed by framework [27] and inductive–deductive hybrid [28] methods. Themes will be extracted to inform the adaptations of NF-Web.

For aim 2, we will test feasibility according to the proportion of participants who agree to participate from those who express interest. Acceptability will be calculated as the number of participants who complete the web program and provide a posttest from those who start the program. We will assess the credibility of the web program using the Credibility Questionnaire (means, SDs, and IQRs) [25]. We will measure satisfaction with the web program using the Client Satisfaction Questionnaire (means, SDs, and IQRs) [24]. We will measure user experience with the User Experience Questionnaire (means, SDs, and IQRs) [23] and qualitative exit interview data. Upon completion, we will explore within-group improvements in NF-Web from baseline to posttest. Our hypotheses are that there will be positive within-group improvements in the secondary outcomes of NF-Web.

For our quantitative analyses, we plan to use linear contrasts to compare changes in secondary (QoL, emotional distress, pain) variables from baseline to posttest within the NF-Web intervention. For QoL outcomes that have established minimal clinically important differences [29], we will consider improvements from baseline to posttest to be clinically meaningful if the mean improvement is above 6.25.

We expect to use SPSS 25 software (IBM Corporation) for the statistical analyses, with an analytic strategy that mirrors the efficacy trial [11]. As our study is a small open pilot trial, we will not be adequately powered to detect within-group or between-groups differences for quantitative outcomes. We will also not be able to examine demographic variables beyond their descriptive aspects. Even so, baseline to posttest will be explored for each outcome in NF-Web, with descriptive statistics and signals of improvement being reported as relevant. If the NF-Web program confirms the primary outcomes (feasibility or acceptability) and trends positively for the secondary outcomes, we will consider this to be preliminary evidence that NF-Web is a useful psychosocial web-based program.

### Data Management

We will store the collected data in a secure location on computers with password protection to maximize confidentiality and security. We will assign each subject a unique, anonymous identifier that is associated with all collected data, including questionnaires and subject-completed logs. Any paper data files will be stored in a secure, locked location that is only accessible to the study team, and these files only include coded subject identification. In addition, all electronic questionnaire data are stored on REDCap [15], a web-based, Health Insurance Portability and Accountability Act–compliant data system available through our academic medical center.

## Results

The NF-Web open pilot clinical trial is ongoing. As of April 2020, we completed the qualitative interviews (n=23) and analyzed the data to construct the first version of the website for our open pilot study. We successfully recruited both cohorts of patients to participate in the trial with the goal of recruiting 30 individuals in total being met (n=24 completed posttest after completing the program). We ended the open pilot trial in May 2021. We aim to complete data analyses by October 2021 and then prepare NF-Web for dissemination in accordance with the results of the fully powered efficacy trial for adults with NF.

## Discussion

### Comparison With Prior Work

Patients diagnosed with NF have a lower but modifiable QoL. Virtual mind–body interventions developed to improve stress management and QoL in people with NF are feasible, acceptable, and beneficial [3,5,6]. However, most patients with NF still struggle to access specialized, evidence-based psychological treatment, even with virtual adaptations. This paper describes the study design and specific strategies used to conduct qualitative interviews with adult patients diagnosed with NF from our ongoing efficacy trial to inform the development of a novel web-based platform to teach resiliency skills and stress management to improve QoL in people with NF. We provide details on the protocol and the potential benefits and challenges of delivering psychosocial care using a web-based platform. We also discuss the methods of monitoring and addressing participant progress and safety throughout the program.

This protocol presents a novel method for delivering care to patients with rare diseases across the globe and provides invaluable information for future trials using asynchronous platforms to deliver psychological care. The web-based program retains the evidence-based mind–body skills associated with improved QoL and psychosocial functioning [11], while providing a cost-effective and scalable delivery modality that could be implemented effortlessly with few human resources. In addition, all program materials could be seamlessly delivered through the website with cumbersome aspects of the live program (eg, homework submission, materials, and resources) found in 1 place. In the future, this program could also be easily translated and culturally adapted for delivery to non–English-speaking patients with NF worldwide. Video content can also be designed to have download capability so that individuals with low broadband connections can store the videos locally on their devices and watch without interruption. Such web-based programs have been developed for other medical populations with a psychosocial profile similar to that of NF (eg, chronic pain, diabetes) [26,27] and have been shown to be feasible, accepted by patients, and in some cases as effective as virtual or in-person interventions [30]. NF-Web represents a potential first-line treatment for patients with NF-specific psychosocial difficulties.

Similar to the DoD trial, we aim to implement and disseminate NF-Web in conjunction with the live video program throughout NF centers across the world, as well as through the Children’s Tumor Foundation (CTF) and local foundations. This program addresses the need for programs that require greater human resources to be addressed by streamlining psychosocial treatment for adults with NF. The web-based treatment model can be used to inform other populations within the NF community (adolescents, families), as well as other medical illness populations.

### Strengths and Limitations

Even with adaptation, there are limitations to consider in the proposed study. First, diverse recruitment poses a barrier to the generalizability of the findings. Previous trials, including our adult efficacy trial, have enrolled mostly middle-class, higher-educated White female patients. We hope that with stronger relationships and involvement from foundations within the NF community, we can maximize opportunities to recruit diverse patients. Although a geographically diverse sample may have its own limitations (eg, differences between geographic regions and major language differences), it also serves as a strength of the program by promoting more flexibility (eg, participants are not bound to a schedule and can participate in their own time) not seen in the live video program. Our use of a geographically diverse sample is consistent with our other NF trials. Second, the live video program is only available in English. Recorded video offers opportunities to provide multilingual subtitles, which can aid program delivery; however, verbal and written materials are currently offered in English only, which limits the individuals who may participate. Finally, although the rationale is to develop a program that can be inclusive of people with barriers to live virtual psychosocial treatment (eg, scheduling, appearance concerns), there are other barriers for participants that NF-Web will not address yet, including participants without technology resources or those with limited financial means.

### Conclusions

Our protocol describes the plans to develop the first-ever stand-alone web-based program to address the psychosocial needs of adults with NF1, NF2, and schwannomatosis. Improvement in outcomes and results related to feasibility, attrition, credibility, and satisfaction will inform implementation of the web-based platform in future clinical trials and other NF populations (eg, adolescents with NF1 and NF2, parents of children with NF1 and NF2, adults with NF2 who are deaf). The results also have the potential to inform adaptations of web-based programs for other medical and non–English-speaking populations.

## Appendix

Multimedia Appendix 1 Peer-review report by the Neurofibromatosis Northeast Medical and Science Committee.

## Abbreviations

3RP-NF: Relaxation Response Resiliency Program for Neurofibromatosis
CRC: clinical research coordinator
CTF: Children’s Tumor Foundation
DoD: Department of Defense
NF: neurofibromatosis
NF-Web: Neurofibromatosis-Web
PI: principal investigator
QoL: quality of life
RCT: randomized controlled trial
REDCap: Research Electronic Data Capture

## References

[1] Vranceanu A, Merker VL, Park E, Plotkin SR (2013). Quality of life among adult patients with neurofibromatosis 1, neurofibromatosis 2 and schwannomatosis: a systematic review of the literature. J Neurooncol.

[2] Wang DL, Smith KB, Esparza S, Leigh FA, Muzikansky A, Park ER, Plotkin SR (2012). Emotional functioning of patients with neurofibromatosis tumor suppressor syndrome. Genet Med.

[3] Vranceanu A, Merker VL, Plotkin SR, Park ER (2014). The relaxation response resiliency program (3RP) in patients with neurofibromatosis 1, neurofibromatosis 2, and schwannomatosis: results from a pilot study. J Neurooncol.

[4] Vranceanu A, Riklin E, Plotkin S, Park E (2015). Psychosocial presentation of adults with neurofibromatosis 1, 2 and schwannomatosis enrolled in a stress reduction program. Proceedings of the Children's Tumor Foundation NF Conference.

[5] Lester E, DiStefano S, Mace R, Macklin E, Plotkin S, Vranceanu A (2020). Virtual mind-body treatment for geographically diverse youth with neurofibromatosis: a pilot randomized controlled trial. Gen Hosp Psychiatry.

[6] Funes CJ, Mace RA, Macklin EA, Plotkin SR, Jordan JT, Vranceanu A (2019). First report of quality of life in adults with neurofibromatosis 2 who are deafened or have significant hearing loss: results of a live-video randomized control trial. J Neurooncol.

[7] Lester E, Macklin EA, Plotkin S, Vranceanu AM (2020). Improvement in resiliency factors among adolescents with neurofibromatosis who participate in a virtual mind-body group program. J Neurooncol.

[8] Greenberg J, Lin A, Zale EL, Kulich RJ, James P, Millstein RA, Shapiro H, Schatman ME, Edwards RR, Vranceanu A (2019). Development and early feasibility testing of a mind-body physical activity program for patients with heterogeneous chronic pain; the getactive study. J Pain Res.

[9] Mace RA, Gates MV, Popok PJ, Kulich R, Quiroz YT, Vranceanu A Feasibility trial of a mind-body activity pain management program for older adults with cognitive decline. Gerontologist..

[10] Mace RA, Doorley JD, Popok PJ, Vranceanu A (2021). Live video adaptations to a mind-body activity program for chronic pain and cognitive decline: protocol for the virtual active brains study. JMIR Res Protoc.

[11] Vranceanu A, Zale E, Funes C, Macklin E, McCurley J, Park E, Jordan J, Lin A, Plotkin S (2018). Mind-body treatment for international English-speaking adults with neurofibromatosis via live videoconferencing: protocol for a single-blind randomized controlled trial. JMIR Res Protoc.

[12] Cohen S, Kamarck T, Mermelstein R (1983). A global measure of perceived stress. J Health Soc Behav.

[13] Cohen S, Spacapan S, Oskamp S (1988). Perceived stress in a probability sample of the United States. The Social Psychology of Health: The Claremont Symposium on Applied Social Psychology.

[14] Kroenke K, Spitzer RL, Williams JB (2001). The PHQ-9: validity of a brief depression severity measure. J Gen Intern Med.

[15] Harris PA, Taylor R, Thielke R, Payne J, Gonzalez N, Conde JG (2009). Research electronic data capture (REDCap) - a metadata-driven methodology and workflow process for providing translational research informatics support. J Biomed Inform.

[16] Skevington SM, Lotfy M, O'Connell KA (2004). The World Health Organization's WHOQOL-BREF quality of life assessment: psychometric properties and results of the international field trial. A report from the WHOQOL group. Qual Life Res.

[17] Spitzer RL, Kroenke K, Williams JB, Löwe B (2006). A brief measure for assessing generalized anxiety disorder: the GAD-7. Arch Intern Med.

[18] Farrar JT, Young JP, LaMoreaux L, Werth JL, Poole RM (2001). Clinical importance of changes in chronic pain intensity measured on an 11-point numerical pain rating scale. Pain.

[19] Keller S, Bann CM, Dodd SL, Schein J, Mendoza TR, Cleeland CS (2004). Validity of the brief pain inventory for use in documenting the outcomes of patients with noncancer pain. Clin J Pain.

[20] Kroenke K, Spitzer RL, Williams JB (2003). The Patient Health Questionnaire-2: validity of a two-item depression screener. Med Care.

[21] Kroenke K, Spitzer RL, Williams JB, Monahan PO, Löwe B (2007). Anxiety disorders in primary care: prevalence, impairment, comorbidity, and detection. Ann Intern Med.

[22] Thompson ER (2016). Development and validation of an internationally reliable short-form of the Positive and Negative Affect Schedule (PANAS). J Cross-Cult Psychol.

[23] Laugwitz B, Held T, Schrepp M, Holzinger A (2008). Construction and evaluation of a user experience questionnaire. HCI and Usability for Education and Work. USAB 2008. Lecture Notes in Computer Science, Vol 5298.

[24] Attkisson CC, Zwick R (1982). The client satisfaction questionnaire. Psychometric properties and correlations with service utilization and psychotherapy outcome. Eval Program Plann.

[25] Devilly GJ, Borkovec TD (2000). Psychometric properties of the credibility/expectancy questionnaire. J Behav Ther Exp Psychiatry.

[26] QSR International.

[27] Gale NK, Heath G, Cameron E, Rashid S, Redwood S (2013). Using the framework method for the analysis of qualitative data in multi-disciplinary health research. BMC Med Res Methodol.

[28] Fereday J, Muir-Cochrane E (2016). Demonstrating rigor using thematic analysis: a hybrid approach of inductive and deductive coding and theme development. Int J Qual Methods.

[29] Vranceanu A, Riklin E, Merker VL, Macklin EA, Park ER, Plotkin SR (2016). Mind-body therapy via videoconferencing in patients with neurofibromatosis: an RCT. Neurology.

[30] Wantland DJ, Portillo CJ, Holzemer WL, Slaughter R, McGhee EM (2004). The effectiveness of web-based vs. non-web-based interventions: a meta-analysis of behavioral change outcomes. J Med Internet Res.

