# Supplementary material for: Adaptation of a Live Video Mind–Body Program to a Web-Based Platform for English-Speaking Adults With Neurofibromatosis: Protocol for the NF-Web Study
Source: JMIR Res Protoc. 2021 Jun 10;10(6):e27526. doi: 10.2196/27526 (PMC8262670; doi:10.2196/27526)
Supplement: Multimedia Appendix 1 [file resprot_v10i6e27526_app1.pdf]

Neurofibromatosis Northeast
Promoting Research, Awareness and Advocacy since 1988

BOARD of DIRECTORS

Lori Ryan, RN, MS, Chair
Patrick Callahan, Esq.
Alexandra Powers Cellucci
Justin Jordan, MD, MPH
Robert Klouda
Andrés Lessing
Melissa Malerba
John Manth
Robert Ryan
John Shea
John Thomson, PhD
Nicole Ullrich, MD, PhD

Karen Peluso
Executive Director

22 February 2021

Dear Dr. Lester,

The Neurofibromatosis Northeast Medical and Science Committee reviewed the MGH research team's proposal entitled, *"In Need and Hard to Reach: Adaptation of an evidence-based Neurofibromatosis resiliency training program for web-based delivery".* The Medical and Science Committee approved the proposal with review and discussion details noted below.

Resume and Summary of Review: The current application for funding proposed to examine the preliminary effects of the evidence-based mind-body program, the Relaxation Response Resiliency Program for Neurofibromatosis (NF), as an asynchronous website-based (i.e., web-based) version of the program called *NF-Web*. The project aims to address an experienced gap in psychosocial treatment for people with NF because of explicit barriers to their care (scheduling, traveling, mental health issues, legitimate functional limitations produced by NF).

Innovation: The specific innovation of this proposal is the adaptation of the program to a self-paced program whereby participants can complete the intervention in their own time. Another innovation is that the applicants have clearly included participants in their trial to learn from their experiences participating in the original 3RP-NF program to aid in making key adaptations for the web-based program.

Design: The overall design of the project is rigorous qualitatively and the open pilot study will likely be able to produce meaningful evidence for future adaptations and implementation of such programs.

Approach: The Approach the applicants take is appropriate specifically because it mirrors previous funded research from their research program (Department of Defense R01 grant; DoD).

Analytic Plan: All measures and Analytic Plans appear to be well thought out and appropriate for the sample size of both the qualitative and quantitative arms of this project. The project seems very feasible and supported by the investigators previous psychosocial clinical research in NF.

Environment: The Environment is top quality and very conducive for such a project given the resources of Massachusetts General Hospital and Harvard Medical School, as well as the Co-PI (Lester) having access to previous participants from the DoD trial as well as exceptional mentorship from Co-PI (Vranceanu).

Weakness: The Weaknesses of the proposed study include limited sample size, the previous difficulties acknowledged in recruiting “hard-to-reach” medical populations, and challenges to recruiting diverse participants for the trial outside the current recruitment list from the DoD trial. It is also unclear at the current time whether participants from the DoD trial would be willing to participate in exit interviews related to their 3RP-NF program experience, however it appears the applicants have a large list of potential participants given the size of this trial.

Summary: Overall, the Neurofibromatosis Northeast Medical & Science Committee are enthusiastic and supportive of the project and endorse the potential positive impact the development of a web-based program could have on both the patient psychosocial experience as well as the overall development of programs for “hard-to-reach” medical populations. Based on these positive marks for the current project, as well as the current budget, funding was approved at $50,000 over 2 years.

Please let me know if you have any questions or need any additional information. Thank you for your research in the NF field!

Regards,

Lori Ryan

Lori Ryan, RN, MS

Chair, NF Northeast Medical and Science Committee

Neurofibromatosis Northeast is a 501(c)(3) tax exempt organization

9 Bedford Street, Burlington, MA 01803 ~781-272-9936 ~ info@nfnortheast.org ~ www.nfnortheast.org
